# Trauma center vs. nearest non-trauma center: direct transport or bypass approach for out-of-hospital traumatic cardiac arrest

**DOI:** 10.1186/s13049-025-01335-0

**Published:** 2025-02-11

**Authors:** Ming-Fang Wang, Chen-Bin Chen, Chip-Jin Ng, Wei-Chen Chen, Shang-Li Tsai, Chien-Hsiung Huang, Chi-Yuan Chang, Li-Heng Tsai, Chi-Chun Lin, Cheng-Yu Chien

**Affiliations:** 1 Department of Emergency Medicine, Linkou and College of Medicine, Chang Gung Memorial Hospital, Chang Gung University, Taoyuan, Taiwan; 2 Department of Emergency Medicine, Chang Gung Memorial Hospital Taipei Branch, Taipei, Taiwan; 3 Department of Emergency Medicine, New Taipei Municipal Tu Cheng Hospital and Chang Gung University, New Taipei City, Taiwan; 4 Department of Nursing, Chang Gung University of Science and Technology, Taoyuan, Taiwan; 5 Present Address: Graduate Institute of Management, College of Management, Chang Gung University, Taoyuan, Taiwan; 6 Department of Nursing, National Taiwan University Hospital, Taipei, Taiwan; 7 Department of Emergency Medicine, Ton-Yen General Hospital, Zhubei, Taiwan; 8 Department of Senior Service Industry Management, Minghsin University of Science and Technology, Hsinchu, Taiwan

**Keywords:** Traumatic cardiac arrest, Trauma centers, Emergency medical services, Cardiopulmonary resuscitation, Transport

## Abstract

**Background:**

Out-of-hospital traumatic cardiac arrest (TCA), a sudden loss of heart function caused by severe trauma such as blunt, penetrating, or other injuries, presents significant public health challenges due to its high severity and extremely low survival rates. Approximately 2.7% of trauma patients experience cardiac arrest at the scene, with an overall survival rate of less than 5%. The correlations of prognosis with various transport approach, such as hospital level with different distance, are yet to be clarified. Thus, we conducted this study to assess the association of transporting TCA patients to hospitals of different levels and distances on critical outcomes, including the return of spontaneous circulation (ROSC), survival to admission, and 30-day survival.

**Methods:**

This retrospective study included adults with TCA who were admitted to various emergency departments in Taoyuan City between January 2016 and December 2022. The patients were stratified by destination hospital into three groups: those transported to a trauma center (TC; TC group), those transported to the nearest non-TC (non-TC group), and those cross-regionally transported to a TC (cross-region TC group). Geographic information system (GIS) data were utilized to determine hospital locations and distances. The associations between various factors and key outcomes—any return of spontaneous circulation (ROSC), survival to admission, 24-h survival and 30-day survival—were analyzed. Multivariable logistic regression was used to determine the association of these outcomes based on transportation to hospitals of different levels.

**Results:**

This study included 557 patients with TCA (TC: 190 [direct transport: 72; cross-region transport: 118]; non-TC: 367). The TC and cross-region TC groups demonstrated significantly higher rates of ROSC at 30.6% and 30.5%, respectively, as well as lower mortality rates (95.8% for both), compared to the non-TC group, which had a ROSC rate of 12.0% and a mortality rate of 99.5%. Multivariable analysis revealed significant associations between favorable outcomes and transportation to a trauma center, either directly (aOR 2.91, 95% CI 1.54–5.49) or via cross-region transfer (aOR 2.05, 95% CI 1.01–4.15). Furthermore, blunt trauma was significantly associated with a poorer survival prognosis (aOR 0.31, 95% CI 0.08–0.78).

**Discussion:**

This study highlights the positive associations of direct or cross-region transportation to a TC on the outcomes of TCA. Our findings challenge the current EMT transport approach in Taiwan, which prioritizes transporting TCA patients to the nearest hospital regardless of its level, potentially leading to worse outcomes. Transport time and TC distance may not significantly influence prognosis.

**Conclusion:**

Bypassing and directly transporting to a TC within the observed (10 km) distances are associated with better survival rates in patients with TCA. Furthermore, blunt TCA is associated with a poorer survival prognosis compared to other mechanisms of trauma-induced cardiac arrest.

## Introduction

Out-of-hospital TCA presents significant public health challenges because of its severity and very low survival rates. TCA is a sudden loss of heart function directly caused by severe trauma, such as blunt, penetrating, or other injuries, leading to the cessation of cardiac mechanical activity. About 2.7% of trauma patients suffer cardiac arrest at the scene, with an overall survival rate of under 5% [1, 2]. Trauma accounts for 8% of all global deaths and is the primary cause of mortality in young individuals [3].

Previous international studies, including a meta-analysis, have shown that the overall survival rate for patients with TCA can range from 1.9 to 8.3%, and in certain circumstances, it can be as low as 1% [4–7]. However, a recent Swedish study demonstrated that the 30-day survival rate of TCA patients transported to a level 1 trauma center was as high as 10.6% [8]. Local data from Taiwan revealed outcomes similar to the global trend. Two studies from Taiwan revealed that 16% to 33.5% of TCA patients had any ROSC, 14.7% were admitted for additional care, 2.1% survived for at least 30 days, and 1.5% survived till discharge [9, 10].

In Taiwan, TCA patients are typically transported to the nearest hospital, irrespective of whether it is a trauma center. However, emergency medical technicians (EMTs) ultimately make the decision, taking into account factors such as the mechanism of the trauma, the medical capabilities of available hospitals, and the preferences of the patient's family. A local study revealed that transportation to a Level I TC was significantly associated with the achievement of sustained ROSC in patients with TCA [11]. However, a prolonged transport time is negatively associated with survival after TCA [7]. Although transport to level 1 trauma centers may take longer, determining how to effectively transport TCA patients to these centers to improve survival rates remains to be explored.

In summary, the relationship between prognosis and transport factors, such as hospital level and distance, remains unclear. The prognostic impact of transferring TCA patients to the nearest non-trauma hospital versus a longer transfer to a trauma center is still uncertain. Therefore, our study aims to investigate the associations of hospital level and distance on the rates of ROSC, survival to admission, 24-h survival, and 30-day survival, also the factors influencing outcomes in patients with TCA.

## Methods

### Study design and setting

This retrospective study included adults with TCA who were sent to various emergency departments across Taoyuan City, northern Taiwan, between January 2016 and December 2022. Taoyuan City is a municipality with a population of approximately 2.3 million and a population density of approximately 1,853 individuals per square kilometer (as of 2022). This municipality encompasses urban, rural, and mountainous areas. The Taoyuan Fire Department (TYFD) has 41 EMS ambulance stations and 1 dispatch center. Taoyuan City has 11 hospitals with the responsibility of providing first-aid; among these, only one hospital is a level I TC—the Linkou Branch of Chang Gung Memorial Hospital (CGMH). EMTs under the EMS system of Taoyuan City hold either intermediate or paramedic certifications. An EMT–paramedic can perform intubation and administer epinephrine or amiodarone through intravenous injection or intraosseous infusion. By contrast, an EMT–intermediate can perform laryngeal mask airway insertion and administer medications through intravenous injection or intraosseous infusion. Mechanical CPR is continuously provided to the patient until arrival at the nearest hospital.

CGMH is a tertiary care hospital that provides comprehensive services, from trauma injury care to rehabilitation. CGMH meets the American College of Surgeons (ACS) criteria and is designated as a Level I trauma center according to the ACS's "Resources for Optimal Care of the Injured Patient" [12]. This designation signifies that CGMH provides the highest level of trauma care, addressing all types of traumatic injuries. The center operates with specialized staff available around the clock and is actively engaged in both research and educational initiatives.

### Participants

All adult (age > 18) patients who suffered from TCA in Taoyuan City between 2016 and 2022 and were transported by emergency medical services (EMS) were included in this study. Patients were excluded if they were pronounced dead at the scene, had incomplete outcome or EMS time data, or if the TCA was due to hanging, as such cases primarily involve airway obstruction and often multiple drug use, differing from our study's focus.

### Data collection and variables

Patients included in this study were divided into three groups on the basis of the destination hospital: the TC, non-TC, and cross-region TC groups. Geographic Information System (GIS) data were analyzed to identify the locations of TCA cases and their distances to TC or the nearest hospitals. TCA patients were classified into the TC group if they were transported to the nearest hospital that is also a Trauma Center. Conversely, they were classified into the non-TC group if the nearest hospital was not a Trauma Center. Cross-region transportation to a TC was defined as the transfer of patients with TCA to a distant TC because the hospital nearest to the event location was a non-TC. Cross-region TC refers to the transfer of patients over a distance to a trauma center, which differs from the TC group where the nearest hospital is also a trauma center.

Prehospital data on patients’ demographic characteristics, event location, trauma mechanism, witness, shockable rhythm on initial cardiac monitoring, bystander CPR, EMS response time, scene time interval, transport time, and prehospital resuscitation duration were collected from the EMS records of the TYFD. In addition, prognosis data on ROSC, 24-h survival, survival to admission, and 30-day survival were collected from the TYFD. Geographic information system (GIS) data were utilized to determine hospital locations and distances. Data (in Chinese) on the locations of traumatic events and those of the nearest and destination hospitals were incorporated into two-degree Universal Transverse Mercator and then projected onto the map of Taoyuan City by using a GIS (QGIS; version 3.30.2). Then, the shortest “real traffic route” from the event location to the nearest and destination hospitals was calculated using QGIS Network Analysis Toolbox. In-hospital emergency procedures include intraosseous (IO) access, Resuscitative Endovascular Balloon Occlusion of the Aorta (REBOA), Emergency Department (ED) thoracotomy, venous cutdown, and extracorporeal membrane oxygenation (ECMO). The distance ratio was defined as the ratio of the distance to a TC compared to the distance to the nearest hospital.

The data that support the findings of our study are available from the first author upon reasonable request (email: tonychen78041801@gmail.com). This study was approved by the Ethics Committee of CGMH (approval number: 202301242B0). The requirement for informed consent was waived because of the retrospective nature of the study.

### Study outcomes

The primary outcome was any ROSC recorded by either an EMT or an emergency physician. The secondary outcomes were survival to admission, defined as patients who survived to be admitted to the intensive care unit (ICU) after undergoing surgery or angiography for embolization, 24-h survival, and 30-day survival (survival for at least 30 days after the traumatic event).

### Statistical analysis

Descriptive statistics are presented as mean ± standard deviation or median [interquartile range] values for continuous variables and the count (percentage) values for categorical variables. A one-way analysis of variance was performed to compare the effect of transport to different hospital levels and distances among the three groups (TC, non-TC, and cross-region TC groups). The Kruskal–Wallis test was performed for data exhibiting a nonnormal distribution. The chi-square test was performed to examine between-group proportion disparities. Logistic regression was performed to determine the association between ROSC, survival to admission, 24-h survival, and 30-day survival based on transport to different hospital levels. Variables identified to be significant (*p* < 0.15) in univariable logistic regression were considered in multivariable models [13, 14]. Statistical analyses were performed using SPSS (version 26.0; IBM Corporation, Armonk, NY, USA). All tests were two-sided. A *p* value of < 0.05 indicated statistical significance.

## Results

**Fig. 1:**
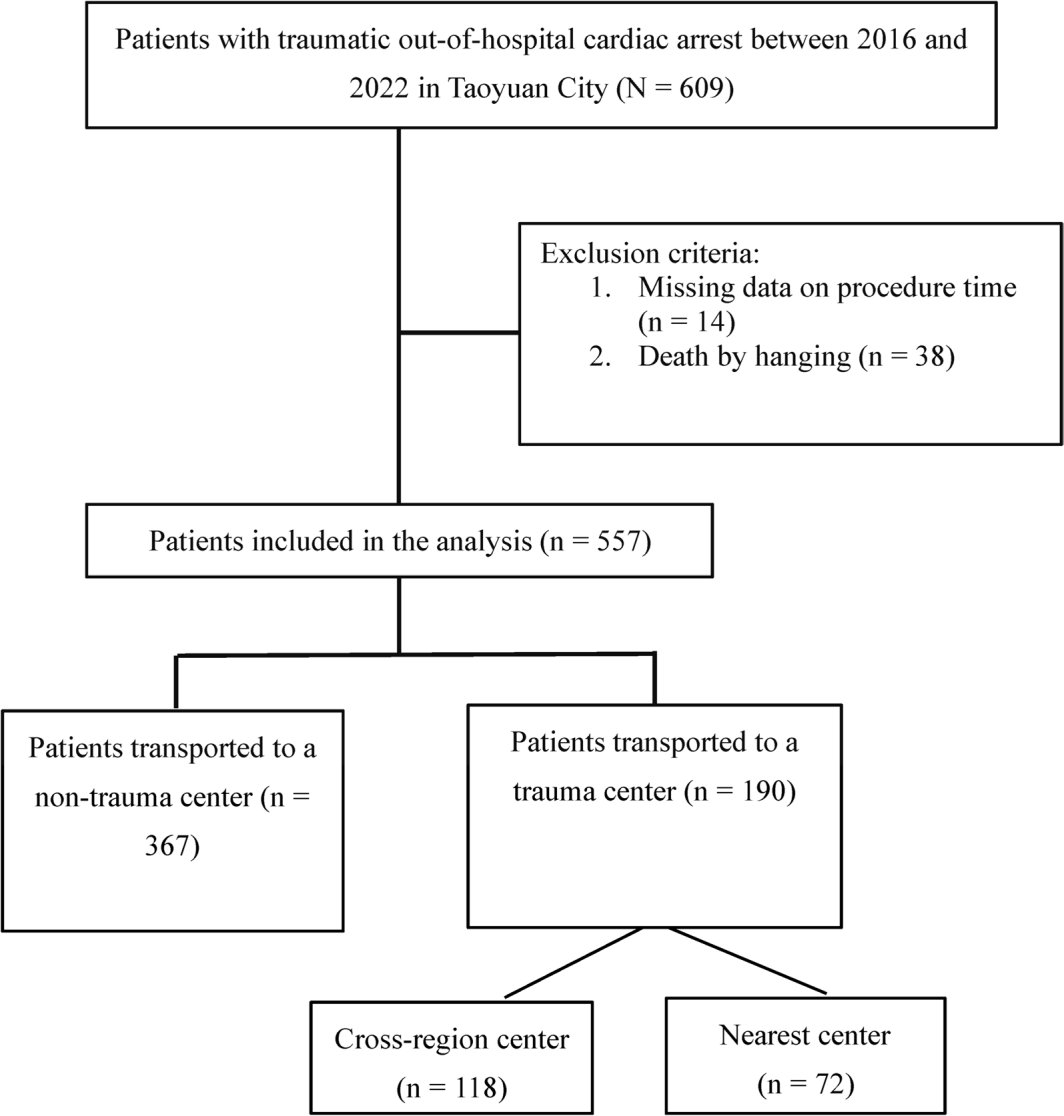
Flowchart depicting patient selection and group allocation

Between 2016 and 2022, 609 patients with TCA were transported to three types of hospitals in Taoyuan City. From these patients, we excluded 38 patients who had committed suicide (only hanging) and 14 patients with missing procedure time data. Finally, our study included 557 patients. The TC and non-TC groups comprised 190 and 367 patients, respectively. Among the patients in the TC group, 72 were transported directly and 118 were transported cross-regionally. Figure 1 presents a flowchart of patient selection and group allocation.

Table 1 presents the characteristics of patients with TCA. Their mean age was 48.6 ± 20.5 years, and 65.7% of them were men. The cross-region TC group was younger than the other groups (*p* = 0.013). Significant between-group differences were noted in both event location and trauma mechanism. The proportion of patients with multiple injuries was higher in the TC group than in the other groups. The predominant trauma mechanism was blunt injury (94.8%), followed by penetrating injury (2.5%) and burn injury (1.8%). The proportion of patients with injuries across body parts (except for abdominal and pelvic injuries) was higher in the TC group than in the other groups; this proportion was higher in the cross-region TC group than in the non-TC group.

**Table 1:** Characteristics of patients with traumatic cardiac arrest

Characteristics	All patients n = 557	Group			*p-value*
		Non-TC n = 367	Cross-region TC n = 118	TC n = 72	
*Age*	48.6 ± 20.5	50.2 ± 20.6	43.8 ± 19.8	48.6 ± 20.2	0.013
< 18 y/o	28 (5.0)	18 (4.9)	6 (5.1)	4 (5.6)	0.701
18–64 y/o	403 (72.4)	260 (70.8)	91 (77.1)	52 (72.2)	
> = 65 y/o	126 (22.6)	89 (24.3)	21 (17.8)	16 (22.2)	
*Sex*					0.220
Female	191 (34.3)	134 (36.5)	38 (32.2)	19 (26.4)	
Male	366 (65.7)	233 (63.5)	80 (67.8)	53 (73.6)	
*Past History*					
Diabetes	23 (4.1)	19 (5.2)	2 (1.7)	2 (2.8)	0.245
Hypertension	37 (6.6)	29 (7.9)	4 (3.4)	4 (5.6)	0.213
Stroke	4 (0.7)	2 (0.5)	1 (0.8)	1 (1.4)	0.425
Heart disease	16 (2.9)	13 (3.5)	1 (0.8)	2 (2.8)	0.426
Chronic kidney disease	7 (1.3)	6 (1.6)	1 (0.8)	0 (0.0)	0.857
Cancer	7 (1.3)	6 (1.6)	0 (0.0)	1 (1.4)	0.508
Mental-related illness	52 (9.3)	41 (11.2)	7 (5.9)	4 (5.6)	0.125
Other Disease	8 (1.4)	5 (1.4)	2 (1.7)	1 (1.4)	0.872
*Injury part*					
Head/Face	184 (33.0)	92 (25.1)	55 (46.6)	37 (51.4)	< 0.001
Spine	55 (9.9)	22 (6.0)	20 (16.9)	13 (18.1)	< 0.001
Chest	151 (27.1)	56 (15.3)	57 (48.3)	38 (52.8)	< 0.001
Abdomen/Pelvic	94 (16.9)	42 (11.4)	33 (28.0)	19 (26.4)	< 0.001
Limbs	168 (30.2)	80 (21.8)	46 (39.0)	42 (58.3)	< 0.001
*Mechanism*					0.005
Blunt	528 (94.8)	354 (96.5)	108 (91.5)	66 (91.7)	
Penetration	14 (2.5)	7 (1.9)	6 (5.1)	1 (1.4)	
Burn	10 (1.8)	6 (1.6)	1 (0.8)	3 (4.2)	
Others	5 (0.9)	0 (0.0)	3 (2.5)	2 (2.8)	

Abbreviations: TC, trauma center

**Fig. 2:**
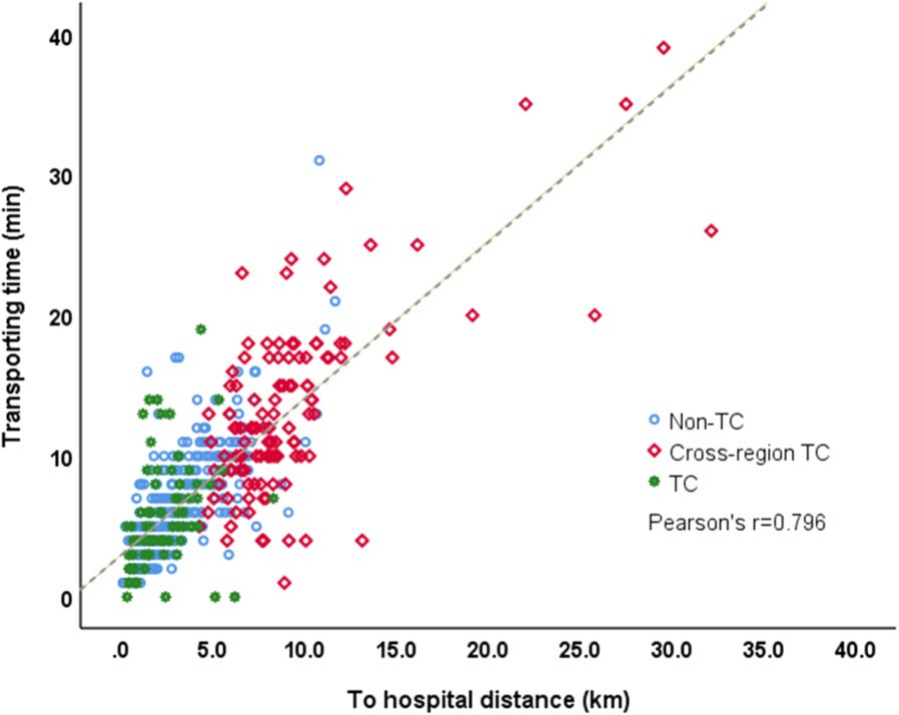
Scatter plot for the correlation between hospital distance and transport time

Table 2 presents information on the primary care and outcomes of TCA. The three groups were similar in terms of the EMS response time (median: 6 min [interquartile range: 4–7]). The transport time was longer—because of longer distances—in the cross-region TC group than in the other groups. A strong linear correlation (Pearson r = 0.796) was noted between hospital distance and transport time (Fig. 2).

**Table 2:** Primary care for and outcomes of traumatic cardiac arrest

Variables	All patients n = 557	Group			*p-value*
		Non-TC = 367	Cross-region TC n = 118	TC n = 72	
Response time median,(IQR)(min)	6 (4–7)	6 (4–7)	6 (4–8)	6 (4–8)	0.826
Scene time interval median,(IQR) (min)	13 (10–18)	13 (10–18)	15 (11–21)	12 (10–17)	0.012
Transport time median,(IQR) (min)	6 (4–10)	5 (4–8)	12 (9–17)	5 (3–8)	< 0.001
Hospital distance median,(IQR) (km)	3.0 (1.6–5.9)	2.3 (1.5–4.0)	8.1 (6.58–10.1)	2.0 (1.2–3.1)	< 0.001
Distance ratio^(a)^	1.0 (1.0–1.2)	1.0 (1.0–1.0)	1.7 (1.27–2.5)	1.0 (1.0–1.0)	< 0.001
Bystander CPR/AED	137 (24.6)	90 (24.5)	30 (25.4)	17 (23.6)	0.960
*Rhythm*					0.008
Non-shockable	553 (99.3)	367 (100.0)	116 (98.3)	70 (97.2)	
Shockable	4 (0.7)	0 (0.0)	2 (1.7)	2 (2.8)	
*Prehospital airway management*					< 0.001
BVM	520 (93.4)	359 (97.8)	107 (90.7)	54 (75.0)	
LMA/Intubation	37 (6.6)	8 (2.2)	11 (9.3)	18 (25.0)	
*Other procedures*					
Neck collar	482 (86.5)	321 (87.5)	103 (87.3)	58 (80.6)	0.281
Chest tube	79 (14.2)	16 (4.4)	41 (34.7)	22 (30.6)	< 0.001
In-hospital emergency special procedures	17 (3.1)	1 (0.3)	10 (8.5)	6 (8.3)	< 0.001
DC shock	31 (5.6)	19 (5.2)	7 (5.9)	5 (6.9)	0.734
Administration of Epinephrine	88 (15.8)	55 (15.0)	29 (24.6)	4 (5.6)	0.002
IV	142 (25.5)	80 (21.8)	53 (44.9)	9 (12.5)	< 0.001
ROSC	102 (18.3)	44 (12.0)	36 (30.5)	22 (30.6)	< 0.001
24-h Survival	53 (9.5)	25 (6.8)	14 (11.9)	14 (19.4)	0.002
*Survival to admission*					< 0.001
Death	479 (86.0)	332 (90.5)	96 (81.4)	51 (70.8)	
ICU	78 (14.0)	35 (9.5)	22 (18.6)	21 (29.2)	
30-day survival	16 (2.9)	4 (1.1)	5 (4.2)	7 (9.7)	< 0.001
*CPC level at discharge*					0.009
1	4 (0.7)	0 (0.0)	2 (1.7)	2 (2.8)	
2	1 (0.2)	0 (0.0)	1 (0.8)	0 (0.0)	
3	3 (0.5)	1 (0.3)	1 (0.8)	1 (1.4)	
4	2 (0.4)	1 (0.3)	1 (0.8)	0 (0.0)	
5	547 (98.2)	365 (99.5)	113 (95.8)	69 (95.8)	

Abbreviations: TC, Trauma Center; CPR, Cardiopulmonary Resuscitation; AED, Automated External Defibrillator; BVM, Bag-Valve-Mask; LMA, Laryngeal Mask Airway; ROSC, Return of Spontaneous Circulation; ICU, Intensive Care Unit; CPC, Cerebral Performance Category. ^(a)^Ratio of the distance of a trauma center to that of the nearest hospital

Among the patients, 102 (18.3%) achieved ROSC either in the prehospital setting or at the hospital. The rate of ROSC was significantly higher in the TC and cross-region TC groups than in the non-TC group. Of the patients, 479 (86%) died upon arrival at the hospital, 55 (9.9%) were admitted to the ICU, 20 (3.6%) were immediately sent to the operation room, and 3 (0.54%) were sent to the angiography room. The mortality rate was significantly (*p* < 0.001) lower in the TC group (70.8%) than in the non-TC group (90.5%) and cross-region TC group (81.4%). Furthermore, the rate of 24-h survival after the event was significantly higher in the TC group (19.4%) than in the non-TC group (6.8%) and cross-region TC group (11.9%). Table 2 presents the patients’ Cerebral Performance Category scores at discharge. Only 10 patients survived to discharge.

Tables 3, 4 and 5 present the correlations of different covariates with any ROSC, survival to admission, and 30-day survival, respectively. The univariable analysis revealed increased probabilities of any ROSC in the cross-region TC group (odds ratio [OR]: 3.22; 95% confidence interval [CI]: 1.95–5.33; *p* < 0.001) and the TC group (OR: 3.23; 95% CI: 1.79–5.84; *p* < 0.001). Advanced airway management techniques (e.g., laryngeal mask airway and endotracheal tube), were significantly associated with an increased probability of any ROSC (OR: 2.62; 95% CI: 1.29–5.35; Table 3).

**Table 3:** Correlations of different covariates with return of spontaneous circulation

Variables	Univariable		Multivariable	
	OR (95% CI)	*p-value*	aOR (95% CI)	*p-value*
*Group*				
Non-TC	ref		ref	
Cross-region TC	3.22 (1.95—5.33)	< 0.001	2.63 (1.55—4.46)	** < 0.001**
TC	3.23 (1.79—5.84)	< 0.001	2.86 (1.52—5.38)	** < 0.001**
Age	0.99 (0.98—1.00)	0.236		
Sex (Male vs. Female)	1.00 (0.64—1.57)	0.996		
Mechanism (Blunt vs. Others)	0.29 (0.13—0.63)	0.002	0.34 (0.15—0.76)	**0.009**
Scene time (min)	1.00 (0.98—1.02)	0.805		
Transport time (min)	1.03 (0.99—1.07)	0.178		
Hospital distance (per 100 m)	1.01 (1.00—1.01)	0.005		
Distance ratio^(a)^	1.04 (1.01—1.07)	0.016	1.02 (1.00—1.05)	0.075
Bystander CPR/AED (Y vs. N)	1.06 (0.65—1.74)	0.817		
Rhythm (Shockable vs. non-shockable)	0.00 (0.00—+ inf)	0.999		
*Prehospital airway management*				
BVM	ref		ref	
LMA/Intubation	2.62 (1.29—5.35)	0.008	1.40 (0.61—3.18)	0.425

Abbreviations: TC, Trauma Center; CPR, Cardiopulmonary Resuscitation; AED, Automated External Defibrillator; BVM, Bag-Valve-Mask; LMA, Laryngeal Mask Airway; ROSC, Return of Spontaneous Circulation; ICU, Intensive Care Unit; CPC, Cerebral Performance Category; aOR, adjusted odds ratio. ^(a)^Ratio of the distance of a TC to that of the nearest hospital. Significant values (*p* < 0.05) are boldfaced

**Table 4:** Correlations of different covariates with survival to admission

Variables	Univariable		Multivariable	
	OR (95% CI)	*p-value*	aOR (95% CI)	*p-value*
*Group*				
Non-TC	ref			
Cross-region TC	2.17 (1.22—3.88)	0.009	1.77 (1.04—3.28)	**0.048**
TC	3.91 (2.11—7.23)	< 0.001	3.36 (1.73—6.52)	** < 0.001**
Age	0.99 (0.98—1.01)	0.394		
Sex (Male vs. Female)	1.05 (0.63—1.75)	0.848		
Mechanism (Blunt vs. Others)	0.28 (0.13—0.63)	0.002	0.32 (0.14—0.73)	**0.007**
Scene time (min)	1.00 (0.98—1.02)	0.798		
Transport time (min)	0.99 (0.95—1.04)	0.805		
Hospital distance (per 100 m)	1.00 (1.00—1.01)	0.200		
Distance ratio^(a)^	1.02 (1.00—1.04)	0.048	1.02 (0.99—1.04)	0.168
Bystander CPR/AED (Y vs. N)	1.15 (0.67—1.98)	0.607		
Rhythm (Shockable vs. non-shockable)	0.00 (0.00—+ inf)	0.999		
*Prehospital airway management*				
BVM	ref		ref	
LMA/Intubation	2.86 (1.35—6.06)	0.006	1.53 (0.65—3.62)	0.330

Abbreviations: TC, Trauma Center; CPR, Cardiopulmonary Resuscitation; AED, Automated External Defibrillator; BVM, Bag-Valve-Mask; LMA, Laryngeal Mask Airway; ROSC, Return of Spontaneous Circulation; ICU, Intensive Care Unit; CPC, Cerebral Performance Category; aOR, adjusted odds ratio. ^(a)^Ratio of the distance of a TC to that of the nearest hospital. Significant values (*p* < 0.05) are boldfaced

**Table 5:** Correlations of different covariates with 30-day survival

Variables	Univariable		Multivariable	
	OR (95% CI)	*p-value*	aOR (95% CI)	*p-value*
*Group*				
Non-TC	ref		ref	
Cross-region TC	4.86 (1.35—17.53)	0.016	4.56 (1.24—16.76)	**0.022**
TC	8.25 (2.27—30.03)	0.001	7.93 (2.13—29.52)	**0.002**
Age	0.99 (0.97—1.02)	0.555		
Sex (Male vs. Female)	1.58 (0.50—4.98)	0.431		
Mechanism (Blunt vs. Others)	0.22 (0.06—0.82)	0.024	0.31 (0.08—0.79)	**0.027**
Scene time (min)	1.02 (1.00—1.05)	0.106	1.03 (0.99—1.06)	0.107
Transport time (min)	1.00 (0.91—1.10)	0.939		
Hospital distance (per 100 m)	1.01 (1.00—1.02)	0.085		
Distance ratio^(a)^	0.98 (0.88—1.10)	0.790		
Bystander CPR/AED (Y vs. N)	1.02 (0.32—3.22)	0.970		
Rhythm (Shockable vs. non-shockable)	0.00 (0.00—+ inf)	0.999		
Prehospital airway management				
BVM	ref			
LMA/Intubation	0.94 (0.12—7.28)	0.949		

Abbreviations: TC, Trauma Center; CPR, Cardiopulmonary Resuscitation; AED, Automated External Defibrillator; BVM, Bag-Valve-Mask; LMA, Laryngeal Mask Airway; ROSC, Return of Spontaneous Circulation; ICU, Intensive Care Unit; CPC, Cerebral Performance Category; aOR, adjusted odds ratio. ^(a)^Ratio of the distance of a TC to that of the nearest hospital. Significant values (*p* < 0.05) are boldfaced

We found no association between any ROSC and factors such as age, sex, EMS scene time, transport time, hospital distance, distance ratio (ratio of the distance of a TC to that of the nearest hospital), or basic life support. Regarding the trauma mechanism, the probability of survival was significantly lower for patients with blunt injury (OR: 0.29; 95% CI: 0.13–0.63) than for those with penetrating injury. (Table 3).

Multivariable models adjusted for the distance ratio and airway management revealed the associations of any ROSC with hospital level and blunt injury. As shown in Table 3, the probability of any ROSC was high in patients cross-regionally transported to a TC (adjusted OR,(aOR): 2.63; 95% CI: 1.55–4.46; *p* =  < 0.001) and those directly transported to a TC (aOR: 2.86; 95% CI: 1.52–5.38; *p* < 0.001) compared to non-TC group but low in those with blunt injury (OR: 0.34; 95% CI: 0.15–0.76; *p* = 0.009) compared to other mechanisms of injury.

As shown in Table 4, the correlations between covariates and survival to admission were consistent with those observed for any ROSC. Survival to admission were consistently associated with cross-region transportation to a TC (aOR: 1.77; 95% CI: 1.04–3.28; *p* = 0.048) or TC (aOR: 3.36; 95% CI: 1.73–6.52; *p* =  < 0.001) compared to non-TC group. These outcomes were associated with blunt injury (aOR: 0.32; 95% CI: 0.14–0.73; *p* = 0.007) compared to other mechanisms of injury.

In the univariable analysis, the 30-day survival rate was higher in the cross-region TC group (OR: 4.86; 95% CI: 1.35–17.53; *p* = 0.016) and the TC group (OR: 8.25; 95% CI: 2.27–30.03; *p* = 0.001) than in the non-TC group. Notably, 30-day survival was negatively associated with blunt injury (OR: 0.22; 95% CI: 0.06–0.82). However, no correlation was found between 30-day survival and prehospital advanced airway management (OR: 0.94; 95% CI: 0.12–7.28). As shown in Table 5, multivariable models adjusted for patient characteristics, trauma mechanism, and scene time interval revealed that the aOR for 30-day survival were 4.56 (95% CI: 1.24–16.76) and 7.93 (95% CI: 2.13–29.52) in the cross-region TC and TC groups compared to non-TC group, respectively.

**Fig. 3:**
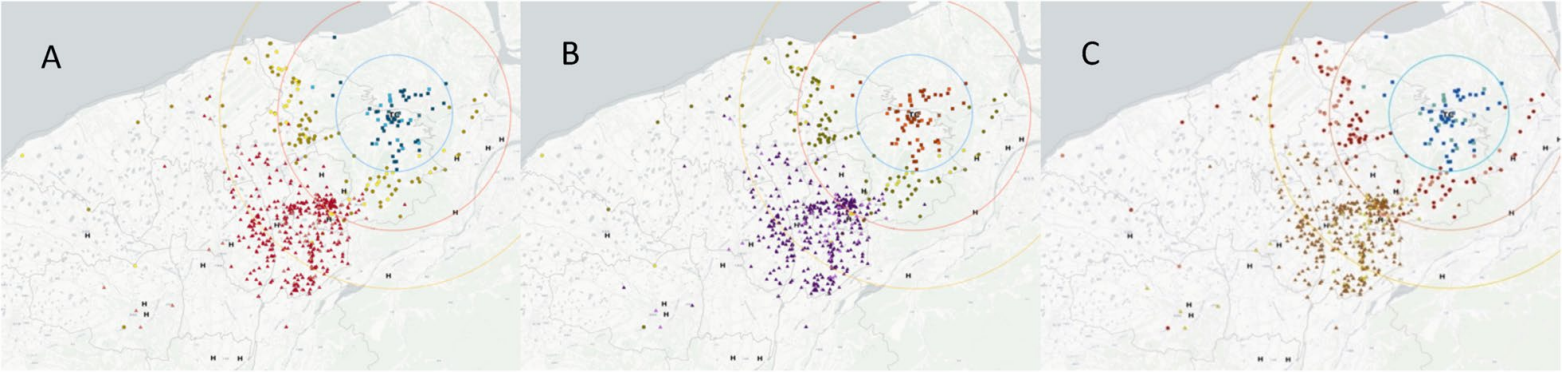
Geographic information system graphics depict the locations of TCA occurrences, nearest hospitals, and level 1 trauma centers **A** any ROSC, **B** survival to admission, and **C** 30-day survival. The square represents the TC group, the circle represents the cross-region TC group, and the triangle represents the non-TC group. Lighter colors indicate any ROSC, survival to admission, or 30-day survival

Figure 3 presents the locations of TCA occurrences, with the circle representing the cross-region TC group, located approximately 10 km from the TC. The three GIS graphics illustrate any ROSC (Fig. 3A), survival to admission (Fig. 3B), and 30-day survival (Fig. 3C) separately.

## Discussion

The overall survival rate after TCA remains low, as demonstrated by our study, which reported a rate of 1.8%. However, the cross-region and TC groups had a higher overall survival rate of 4.2%. The cross-region TC and TC groups exhibited a higher probability of any ROSC, improved survival to admission, and increased 24-h and 30-day survival rates. TC distance and transport time showed no significant association with survival prognosis. However, blunt injury–induced TCA was associated with poor prognosis.

The overall survival rate in our study closely aligns with the reported rate of 1.5% from a single trauma center in a study conducted in southern Taiwan from 2014 to 2016 [9]. However, when patients were transported or bypassed to TC, the overall survival rate was 4.2%, more than eight times higher than that of the non-TC group. Over the past decade, the survival rate for patients sent to TC has increased significantly. In contrast, the Pan-Asian Resuscitation Outcomes Study, conducted across 13 Asian countries, reported a survival to discharge rate of only 3.4%, regardless of hospital level [15].

Regarding survival to admission, previous studies have reported rates ranging from 16.5% to 18% [16, 17]. Our study presents similar findings, with any ROSC rate and survival to admission at 18.3% and 14%, respectively. However, in the TC group, the rates of any ROSC and survival to admission were significantly higher, at approximately 30.6% and 29.2%, respectively. In the cross-region TC group, the ROSC rate and survival to admission were also higher, at 30.5% and 18.6%. After adjusting for covariates, we found that both direct and cross-region transport to a TC were associated with improved outcomes in TCA patients compared to those in the non-TC group. A study conducted in Toronto, which aligns with our findings, indicated that the 24-h and 48-h mortality rates were lower in patients transported directly to a trauma center compared to those transported to a non-trauma center [18]. Another study indicated that the rate of mortality (including in-hospital mortality) after severe trauma was significantly lower for patients transported to a TC than for those transported to a non-TC [19]. In a Swedish study, the adjusted rate of 30-day mortality was 41% lower in patients transported to a TC than in those transported to a non-TC, especially for critically ill patients [20]. However, groups of these three papers were for all trauma patients, not just TCA patients, which is different from ours.

In contrast, some studies have suggested transporting TCA patients to lower-level hospitals. U.S. studies have shown that, after adjusting for confounders such as patient demographics, injury patterns, and Injury Severity Score, the survival rate of TCA patients was higher at Level II hospitals compared to Level I hospitals [21, 22]. However, the authors of these studies noted that performance improvement programs and regionalized trauma care could be significant predictors of survival. Neither study mentioned factors influencing survival outcomes, such as the distance to the destination or proximity to nearby medical institutions.

We found that the probability of overall survival was the highest for patients directly transported to a TC, followed by those with cross-region transportation to a TC and transportation to a non-TC. Similar trends were observed for survival to admission and 30-day survival rates. A study in southern Taiwan reported a significant association between transportation to a TC and the achievement of sustained ROSC in patients with TCA [11], which aligns with our findings. However, that study did not account for distance or proximity to nearby medical facilities. To the best of our knowledge, the present study is the first to report that cross-region transportation to a TC, despite requiring more transport time (a median of 7 min in our study), improves the probability of survival.

In Taiwan, the current protocol for transporting patients with TCA is to the nearest hospital, regardless of its level. Our findings challenge this practice. Once ROSC is achieved, TCA patients require care from a multidisciplinary team, which is only available in Level I TCs. While transport to a TC increases the probability of survival, the time required for transport plays a critical role in patient outcomes. A Taiwanese study reported a negative association between prolonged transport time and survival in patients with TCA [17]. Additionally, several studies have shown that a response time of over 8 min has no significant effect on the survival rate following trauma [7, 23]. In a relevant study, the probability of transportation to a TC decreased by 5% with a 1 km increase in the distance of the nearest TC from the TCA location; this finding suggests that the hospital distance, rather than the injury pattern, is a crucial factor influencing transportation decisions [24]. In the present study, the median transport time was 12 min (interquartile range: 9–17 min) in the cross-region TC group, while it was 5 min in the non-TC group. The longest transport time recorded in our study was 42 min. Despite the extended transport time, our findings indicate that directly transfer to a TC over a longer distance result in better survival probabilities, and the transfer distance had no significant impact on survival outcomes. Thus, considering the convenience and intensity of EMSs in Taiwan, we recommend transporting patients with TCA to a TC with a relatively short transport time. However, the optimal approach may be different for countries with limited EMS or a vast territory.

Many factors might influence the outcomes of TCA; these factors include injury pattern, cardiac arrest recognition, bystander CPR, prehospital intervention, transportation strategy, and hospital care. Some studies show that trauma mechanisms are not associated with ROSC and survival rate [25, 26], but some think that blunt injury is associated with poor outcomes [5, 6]. However, our study findings indicate that blunt injury leads to poor outcomes after TCA. Notably, no association has been observed between in-hospital mortality and pre-hospital advanced airway management, age, or bystander CPR [27].

### Limitations

The present study has some limitations. First, this was an observational, retrospective study. EMT–paramedics made transportation decisions considering real-time factors. These decisions might have been influenced by factors such as age and trauma mechanism. This might have introduced a selection bias in this study. Nevertheless, we attempted to correct this through multivariable analyses. Future studies should adopt a prospective design, as the current study is based on retrospective data, where patient transport decisions by EMTs may introduce bias. By implementing a prospective study design with a standardized transport protocol for EMTs to follow, the risk of bias can be minimized.

Second, Taoyuan City has a high population density and hosts numerous hospitals. In this study, we did not consider the effects of traffic congestion, day–night variations, and holidays, particularly in rural and low-density areas. Thus, our results should be interpreted with caution.

Finally, we lacked data on the patients’ anamneses, which might have influenced our results pertaining to prognosis. Nonetheless, most of the patients were young adults with relatively good health conditions.

Despite the aforementioned limitations, we recommend cross-region transportation to a TC for patients with TCA in high-density population area.

## Conclusion

Bypassing and directly transporting to a TC, within the distances observed in our study, are associated with elevated ROSC rates, improved survival to admission, and increased 30-day survival rates in patients with TCA.333.

## Data Availability

The data that support the findings of our study are available from the first author upon reasonable request (email: tonychen78041801@gmail.com).

## Abbreviations

CPR: Cardiopulmonary resuscitation
CGMH: Chang Gung memorial hospital
EMS: Emergency medical service
EMTs: Emergency medical technicians
GIS: Geographic information system
ICU: Intensive care unit
OR: Oddi ratio
ROSC: Return of spontaneous circulation
TC: Trauma center
TCA: Traumatic cardiac arrest
TYFD: Taoyuan fire department
